# The Odontological Society of Great Britain

**Published:** 1889-05

**Authors:** 


AMERICAN JOURNAL

—OF—

DENTAL SCIENCE.

Vol. xxiii. Third Series—MAY, 1889. No. 1.

ARTICLE I.

### THE ODONTOLOGICAL SOCIETY OF GREAT BRITAIN.

An ordinary meeting of the above Society was held at its Rooms, 40, Leicester Square, on March 4th, Mr. Henry Sewill (President) in the chair.

After balloting for several resident and non-resident members, and the adoption of the Librarian's Report—

Mr. Howard Mummery related a somewhat curious case. A young lady, aged 14, wearing a lower regulation plate, came to him in January to have it adjusted. She had felt something come loose in her mouth, like a piece of gum. On examination, he perceived what was apparently a small pendulous piece of gum tissue about half-an-inch long, hanging from the alveolar margin of the upper jaw over the position of the unerupted left second molar, which caused considerable discomfort. He divided it with a pair of scissors, but at the end he discovered a small nodule of calcified tissue, in shape very much like an acorn. The stalk of the nodule consisted histologically of the ordinary elements of gum tissue, and was firmly adherent to a loose capsule or sac, which, in its turn, was continuous with the calcified nodule, and formed—with an elevated ring of calcified tissue surrounding the nodule—the cup of the acorn, the rest, or acorn part, being a rounded mass of calcified tissue. After making drawings in different positions and sections, he came to the conclusion that the detached portion of gum was an odontome, connected developmentally with the tooth papilla and follicle, no enamcl being present, but only dentine and cementum. Its position was somewhat puzzling, as it was difficult to understand how it came to be connected with the pedicle in the peculiar manner shown in the diagram, and why it became detached. It was apparently liberated by the commencing eruption of the second molar, the outer third of the crown of which was distinctly visible a week after. So far as the structure of the second molar could then be seen, it was a perfectly normal tooth. He believed it was rare to find these abnormal growths connected with the upper teeth in man. Later, the patient described a similar nodule having become detached, but unfortunately it had, in all probability been swallowed, as there was no trace of it.

Mr. W. A. Maggs showed a good specimen of intrinsic calcification of the pulp in a left lower wisdom tooth. The calcification was not quite complete towards its superior surface, and it might fairly be called a "pulp stone." The tooth was much decayed; a severe and persistent neuralgia of that side of the face led to its removal. Mr. Maggs presented the specimen to the museum.

Mr. Ackery and Mr. F. J. Bennett expressed their opinion that the condition was one of investigation of dentine, and not of secondary deposit. Thereupon the President asked Messrs. Maggs, Ackery and Bennett to state their decision upon the matter at the next meeting of the Society.

Mr. Hugh Lloyd Williams showed and described a calculus extracted from the mouth of a strong healthy man, aged twenty-eight. The patient complained of a small swelling beneath the angle of the left jaw which increased to several times its original size and became very painful on the sight or even smell of any savoury food. He had suffered for four years, and, after consulting medical men and having visited one hospital, Mr. Williams saw him. To test the patient's statement as to the swelling, an apple was given him, and even at the sight of it the swelling commenced and continued to increase while it was being eaten. The only apparent sympton on inspection was that the left sub-lingual gland was more prominent than the right; careful bimanual examination indicated hardness in the floor of the mouth, but on account of the inflammatory thickening of the surrounding tissues, it was impossible to clearly define the limits of the calculus. The calculus was ultimately removed with small dissecting forceps. Its direction and shape induced Mr. Williams to think that the growth of these calculi was similar to that of calculus on the teeth.

### DISCUSSION ON DENTAL ANTISEPTICS.

Mr. Robt. H. Woodhouse, after a few introductory remarks, said that his endeavour would be briefly to allude to recent discoveries in the field of antiseptics as they bore upon dental practice. With reference to special localised treatment of the teeth, too great stress could not be laid on the general treatment of the oral cavity. The environment of the teeth was all important; the most skilful operations often failed from neglecting some simple general treatment which tended to counteract a septic condition in and about the teeth. The patient needed to be educated to co-operate with the dentist in getting the best results in this respect. A true antiseptic had been defined as that which prevented putrefaction by arresting or destroying the growth of organisms or the chemical activity of certain substances which gave rise to fermentation or decomposition. The patent fang of a tooth with its surroundings of high temperature, food and water supply, formed an excellent incubator of bacteria, and when neglected would be very much like a minute tropical swamp. With proper treatment, however, a condition could soon be produced which would prevent further multiplication of the bacteria by removal of the pabulum from which they were nurtured. He was not prepared to say how far the micro-organisms extended up the dentinal tubuli beyond the limit of decalcified bone, but he believed they did so extend. In dental operations it was important not only to use every means to destroy micro-organisms, but also to produce a condition of the mouth which left no resting place for them. Whether contour fillings or free divisions were adopted, this should be equally borne in mind. Professor Miller, of Berlin, placed the relative powers of the following germicides in this order:—

| | | |
|---|---|---|
| Perchloride of Mercury — — | 1 in | 100,000 |
| Peroxide of Hydrogen — — | 1 " | 50,000 |
| Iodoform — — — — | 1 " | 6,000 |
| Salicylic Acid — — — | 1 " | 2,000 |
| Eucalyptus Oil — — — | 1 " | 1,600 |
| Carbolic Acid — — — | 1 " | 1,500 |
| Oxide of Zinc — — — | 1 " | 1,250 |
| Permanganate of Potash — | 1 " | 1,000 |
| Listerine — — — — | 1 " | 120 |

To this Mr. Woodhouse added boric acid, which in the form of boroglyceride he found most useful in disease of the antrum or as a mouth wash. The red iodide of mercury, soluble in an excess of iodide of potassium, he stated was considered at least twice as powerful as the perchloride of mercury. Oxide of zinc in proper combination with carbolic acid, Mr. Woodhouse used, and regarded as of the greatest value in the conservative treatment of the dental pulp. He also used this preparation in the case of badly decayed teeth of children where the nerve was not exposed. His custom was to remove the greater part of the decay at the first sitting and place a thick layer of the oxide and carbolic acid formed in a thin paste over the floor of the cavity; this was left for a week or so, when the tooth could be more carefully excavated and filled with Sullivan's cement or osteo. He used the same preparation in adult teeth where exposure had taken place, when it had been decided that the nerve was presumably healthy and would warrant conservative treatment. Although, no doubt, it was sometimes better to leave a little impoverished bone over the nerve rather than expose it, he was, even with the aid of antiseptics, chary of leaving decay in such circumstances. Absolute isolation from the fluids of the mouth alone would stay the progress of decalcification. His experience was that where the dentine had been invaded by decay, it must be removed by polishing or stopping, as no antiseptic would avail if the fluids of the mouth still had access. Mr. Woodhouse described with some detail his own method of dealing with cases of severe toothache and disorganised nerve structure. He used the preparation of oxide of zinc and carbolic acid for filling roots. The larger part of the root canal and nerve cavity, when not used as a retaining point for anchoring the filling of the crown, he filled with chloroform and gutta-percha. If anything then went wrong with the filling the fang remained healthy. He believed that preparations of mercury were most useful in commencing the treatment of root canals in dead and neglected teeth. As a deodorant, after carefully opening up and washing out the nerve canal, he used an application of eucalyptus oil.

The President: I am sure that the members of the Society will agree that Mr. Woodhouse has done exactly what was needed. He has given us a very suggestive paper, and touched upon many points for discussion. The subject of antisepticism is one which I think may well be taken for discussion periodically by the Society. New agents, new facts with reference to their effects, and new points with regard to pathology, are being continually brought forward, and I think that it would be well if for some years we were to devote, say, one evening each Session to this important subject. My contribution will be to bear testimony to the value of perchloride of mercury as an antiseptic agent. The objections which exist to the use of this drug in *general* surgery do not apply to *dental* surgery. In treating the teeth we only use a few drops of the solution, and there is no danger if the whole were swallowed, or any serious constitutional effects. Before going any further, I must express the hope that the discussion will be confined strictly to the point; we do not want to go into qeestions of pathology or diagnosis, and shall do well to keep strictly to the lines laid down by Mr. Woodhouse. The preparation of perchloride of mercury that I use is that dissolved in absolute alcohol, and practically you may use two or three grains to the ounce. Both Professors Koch and Pasteur have proved that perchloride of mercury is vastly more powerful than any other antiseptic we possess, It will destroy germs by the application of one weak solution. In ordinary cavities washing them with one weak solution will make them aseptic. In the case of putrid teeth one washing with a strong solution will act like magic. Of course, in clearing canals one must be careful not to force *debris* through the apical foramen. In dressing root canals, one strong solution of perchloride of mercury in a solution of alcohol is very often enough, though of course it may be repeated over and over again. In conjunction with this I have used iodol, carried in with cotton wool, charged with as much as it will hold. In permanent root fillings I use the same thing, applying the hot air syringe as I go on. Before using perchloride of mercury I used oxychloride of zinc, but since the introduction of the former I have followed the course I have stated. I have been in practice, I am sorry to say, a large number of years now, and I have had sufficient success, in spite of the fact that I am not a good operator, to assure me of the soundness of my method, and of the use of perchloride of mercury as an antiseptic agent in dental surgery.

Mr. F. J. Bennett was especially glad to hear Mr. Woodhouse's remarks with regard to capping the pulp. It had always appeared to him in exposure of the pulp, that in many cases before the pulp is reached the dentine is found to be disorganized. In his own experience he had found that the plan Mr. Woodhouse advocated, or a similar one, had always been most successful. His colleague, Mr. Forsyth, had used a paste of oxide of zinc and oil of cloves, and capping over had finally filled the tooth with osteo. With regard to drugs that are favourities, he knew that perchloride of mercury is the most powerful known, but he thought it desirable to distinguish between a good germicide and what suits one's particular case; it seemed to him that in using it on a piece of cotton wool it must be pretty well evaporated. He had found a very valuable use for perchloride of mercury—and that, by the way, Mr. Woodhouse had not referred to—in sterilizing instruments. He had used eucalyptus oil, and latterly iodol; the value of the latter, he believed, depends upon the amount of iodine set free in the canal.

Mr. Mitchell said with regard to liberation of iodine from iodoform, he applied it to the cavity on cotton wool, and vaporized it with a hot air syringe—which had rather a large opening—heated over a bunsen burner.

The President said that he should have remarked that absolute alcohol has the effect of desiccating the particles which on a second swabbing take up the solution and become sterilized.

Mr. W. J. England was indebted to Mr. Hern some four and a half years ago for the recommendation of perchloride of mercury as a dressing. Cotton wool saturated with it could be earried to the canal quite well on a Donaldson. Mr. Bennett need not fear its drying too quickly; it would take two minutes at least. Mr. England used a strong solution of 5 grains to 1 oz.; on evaporation taking place, perchloride of mercury is left in the form of a fine powder on the canal walls, which may then safely be left to argue the question with any bacteria that may happen to come that way. He found iodol less objectionable in taste or smell than iodoform, and he was not afraid of any irritation.

Mr. W. Hern thought that Mr. Woodhouse struck a very good note, when he said, remove the coign of 'vantage for germs to linger, and also remove the pabulum of the micrococci. The best antiseptic, he thought, is a little rotating drill and the wet syringe—what he might call the hydropathic treatment. Antiseptic drugs should only be used for the removal or purification of the very small portions of septic material that are left. After the greatest possible amount had been removed with the drill, the cavity should be washed thoroughly out. Mr. Hern took exception to the use of essential oils, there being no affinity between them and water, and in septic conditions moisture was very largely present. By way of illustration, he mentioned having taken some micrococci and put them into a globule of eucalyptus oil; under the microscope, these micrococci could be seen, *not in* the globule, but *on the margin of it* enjoying themselves. Perchloride of mercury has a strong affinity for water, and therefore robs the micrococci of one element of their existence, viz., moisture. He still continues to use iodoform in which he has great faith.

Mr. Betts (Hon. Sec.) read a letter from Mr. Charters White stating that had he been able to bc present, he would have brought forward an antiseptic known as "Kingzett's Bactericide." Mr. Betts mentioned that he used *powdered* iodoform mixed in with the wool.

Mr. J. T. Browne Mason (Exeter), had also used dry iodoform in the same way, and found it most satisfactory. It also admits of the advantage of being withdrawn readily if necessary. There was one point he would like explained. In a recent case his patient, for whom six months previously he had put in a successful gold filling, complained of a taste of iodoform; there being no opening of any kind he was at a loss to account for it.

Mr. C. S. Tomes observed that one speaker after another spoke of iodoform setting free its iodine. He did not see why it should; but with iodol it was different, for the latter drug was easily decomposed, and had a tendency to stain the tooth structure.

Mr. Ashley Barrett remarking upon the President's considerable experience, asked how he dealt with those adanced cases where the bacteria had affected the dentinal tubes—having extended into the walls of the canal?

The President's recollection was that Mr. Arthur Underwood has shown, in his valuable research on caries, that micro-organisms *do* penetrate the tubes before they become dilated by the changes of caries, but he (the President) had not investigated that part of the subject. He was himself satisfied that frequent swabbing with any powerful germicide, like perchloride of mercury, and dressings with iodol, would stop all septic action in the canals.

Mr. Boyd Wallis thought that he was the first to call attention to iodoform and iodol as useful dental preparations. His experience was that they are very readily absorbed and very evanescent. He endorsed Mr. Hern's remarks as to essential oils. Eugenol he believed to be the best anodyne we possess, but latterly he had successfully used a solution of sulphur in ozonic ether as a capping for exposed pulps. He used various preparations of naphthol—a bi-product of coal tar—but preferred beta naphthol, which he considered the best antiseptic for dental purposes. It was said to be poisonous, but he had never found it so, although he had used it freely for dusting ulcers.

Mr. Hern remarked that, so far from finding iodoform evanescent, he had recently taken out a filling put in during 1883, and found the odour of iodoform as powerful as ever.

Mr. Geo. Cunningham was of opinion that (in the large majority of cases of root filling) the dental engine is the most important agent in dealing with septic areas, and deprecated the unnecessary resort to antiseptic drugs.

Mr. Walter H. Coffin found oxide of zinc and glycerine, with 1 per cent. of arsenic, extremely good as a root filling. In the case of ramified alveolar abscess he knew of nothing better than peroxide of hydrogen. Kingzett's preparation, with peroxide of hydrogen, was very satisfactory, and it had occurred to him to combine with it perchloride of mercury; in that way, when the oxygen had decomposed, a little of the perchloride of mercury would remain deposited.

Mr. Bland Sutton read a paper on

### THE RELATION OF RICKETS TO SOME FORMS OF ODONTOMES.

Some years ago, he said, he had endeavoured to point out that the delay in the appearance of the teeth in rickety children must be attributed to abnormal thickness of the follicle, and he thought that it should not be a matter of surprise that the tooth-sac should be thickened in rickety children when it was remembered that this remarkable disease affects more particularly those membranes which are engaged in the production of bone. So also would it be inferred that as the tooth-sac was responsible for the cementum of the tooth and a part at least of its alveolus, the follicles in mammals, including rickety children, would be abnormally large. Mr. Sutton pointed out that in nearly all the specimens of fibrous odontomes which he had shown to the Society, the skeleton of the mammal possessing them was severely affected with rickets; in each case the tumours were multiple and symmetrical; those which occured in the upper jaw were much larger than those found in the mandible. Histologically they were composed of young fibrous tissue with laminæ of well-formed bone interspersed, and presenting here and there giant cells. In many cases the fangs of a well-formed tooth were imbedded in the tumour in the upper jaw, but this was rarely so in the lower jaw. Mr. Sutton supported the contention that the association of rickets and fibrous odontomes was not casual, by citing among others the case of two young Assyrian bears killed in the Zoological Gardens because they were deformed by rickets; the follicles of the molar teeth were greatly enlarged, and projected as rounded fibrous tumours. He also referred to two tumours in the collections of the Royal College of Surgeons, which are described as myeloid tumours, and were removed from a boy aged 7½, suffering with rickets. Mr. Sutton had no hesitation in pronouncing these specimens to be overgrown tooth follicles, due to rickets. He remarked that it should be borne in mind that a few myeloid cells are not sufficient to constitute a myeloid sarcoma; the giant cells should make up a very large part of the tumour. In conclusion, while it was far from his intention to insist that all odontomes are to be attributed to rickety changes in the follicles, it seemed probable that rickets was responsible for some both hard and soft varieties; nor need it be a matter of surprise that tooth follicles, enlarged and thickened by rickety changes, might become in later life hard odontomes, seeing that the long bones in the progressive stage of rickets are very soft, and thicken and become hard in cases of recovery.

The President: Mr. Bland Sutton may be said to be a scientific explorer. His valuable and interesting papers here, and his lectures at the Royal College of Surgeons, embodying as they do the results of his investigations, invariably contain much that is new to us, and there is not the least doubt that valuable generalizations will arise from his studies. We all recognize Mr. Bland Sutton as an important member of the scientific world. But, because his papers either contain fresh discoveries or throw entirely new light upon our present knowledge, we find it somewhat difficult to discuss them without time to digest them. There are, however, many members of our society who are capable of discussing them and I hope we shall have a good discussion to-night.

Mr. C. S. Tomes: I believe, Sir, that the observation that Mr. Bland Sutton has made is the outcome of several years' reflection and investigation. I recollect some questions he asked me some years ago, as to alterations that take place in tooth follicles in connection with rickets. This observation is an absolutely original one; I do not remember anywhere to have seen any allusion of the kind, and it is one the value of which to pathology is very obvious. There is one matter that I should like to make a remark upon: Mr. Sutton says that he has carried us a little further in point of classification, and that the specimen would form what we understand by an odontome. Looking at the specimen it does not appear to me that it would. It seems to me, looking at the specimen, that the tooth tissue, as far as the dentine and enamel are concerned, is unaltered, and that had further calcification gone on the result would have been hypertrophy of of cementum. Odontomes resemble only partially the ordinary tooth form, but the specimens handed round are quite normal in form, so that being further calcified the tumours would not correspond quite to any form of odontome that we are familiar with. With regard to Mr. Mummery's specimen, it is a very curious little body. I had the opportunity of seeing it when it was fresh, and it is a little difficult to understand how it erupted. There is one point to which Mr. Mummery has not called attention—though it was merely an oversight on his part, as he remarked it when showing the specimen to me—and that is that its pedicle is attached to the wrong end, being attached to the base of the pulp. I have nothing to say in explanation of the fact, but it is a peculiarity that if thought out may enable one to get at an intelligible history of it.

Mr. Mitchell: I should like to ask Mr. Sutton if he considers this a special class of odontomes, *i. e.*, out and beyond what we ordinarily understand odontomes to be.

Mr. F. J. Bennett: I suppose Mr. Bland Sutton would call this a cementoma? The thickening of the cementum would not alter the formation of the dentine in any way.

Mr. Storer Bennett: I had not the opportunity of seeing the specimen when it was being passed round, but without wishing to challenge Mr. Bland Sutton's description of classification—for that we all know is an extremely dangerous thing to do—I should like to say that there was one point which struck me as rather peculiar. In most animals odontomes occur symmetrically, though, if not absolutely unknown, they are extremely rare in human beings. It seems to me that the question suggests itself that if these odontomes are due to rickets, why do we not get a greater number of symmetrical odontomes in rickety people?

Mr. Paterson: I should like to ask Mr. Sutton if it is necessary for the ordinary sarcoma to contain 4-5ths of myeloid cells in every case?

Mr. Bland Sutton in reply said: Sir, Mr. Tomes is quite right when he remarks that this thing has been slumbering in my mind for some years. I think it is about eight years ago that I had the extreme pleasure of making Mr. Tomes' acquaintance, and it was over the jawbone of a rickety baboon. I asked him several questions, but Mr. Tomes, in that cautious way which should distinguish all true scientific investigators, kept an open mind upon the subject. I have been at work on the relation of rickets to odontomes since that time. Now, odontomes which occur in animals are of two great classes, those which I call cementomata—which are due to the ossification and enlargement of the follicle; and composite odontomes—due to aberrations of the whole tooth germ. To prove that they really arise from such conditions, I had the opportunity of examining the largest that has been known in a horse. I decalcified it, and in the middle of the soft matter there was the outline of three teeth. With regard to the w-shaped odontomes that we find in the human subject, they occur most commonly in the lower jaw; but in the classical case of Hare of Limerick, the odontome occurred in the upper jaw, and had the well formed crown of a tooth attached to it. When soft odontomes occur in the lower jaw of animals they do not contain any trace of tooth substance.

Had the soft tumours in the bears become calcified, we should have had a well shaped tooth, such as we see in the human subject when the odontomes occur in the upper jaw; but in the case of the lower jaw the odontomes would have presented themselves as a shapeless mass of dentinal tissue. With regard to the number of giant cells necessary to make a myeloid sarcoma, if our classifications are to be worth the paper they are written upon, our definitions must be sharply drawn, and therefore it is desirable to say that there must be at least 4-5ths of these myeloid cells, and the tumour should be a maroon colour to make a myeloid sarcoma. It may be interesting to Mr. Paterson to know that the larger the number of these cells the less likelihood there is of a recurrencc of the tumour.—*The Dental Record.*

